# Neoadjuvant therapy of BRCA1-driven ovarian cancer by combination of cisplatin, mitomycin C and doxorubicin

**DOI:** 10.1186/s13053-021-00173-2

**Published:** 2021-02-03

**Authors:** Tatiana V. Gorodnova, Anna P. Sokolenko, Khristina B. Kotiv, Tatiana N. Sokolova, Alexandr O. Ivantsov, Konstantin D. Guseynov, Ekaterina A. Nekrasova, Olga A. Smirnova, Igor V. Berlev, Evgeny N. Imyanitov

**Affiliations:** 1 N.N. Petrov Institute of Oncology, 197758 Saint-Petersburg, Russia; 2 St.-Petersburg Pediatric Medical University, 194100 Saint-Petersburg, Russia; 3 I.I. Mechnikov North-Western Medical University, 195067 St.-Petersburg, Russia

**Keywords:** Ovarian cancer, BRCA1, Neoadjuvant chemotherapy, Cisplatin, Mitomycin C, Doxorubicin

## Abstract

**Background:**

Cisplatin, mitomycin C and anthracyclines demonstrate high activity in BRCA1-deficient tumors. This study aimed to evaluate the efficacy of the triplet combination of these drugs in BRCA1-driven high-grade serous ovarian carcinomas (HGSOCs).

**Methods:**

Ten HGSOC patients with germ-line *BRCA1* mutation received neoadjuvant chemotherapy (NACT) consisting of mitomycin C 10 mg/m^2^ (day 1), doxorubicin 30 mg/m^2^ (days 1 and 8) and cisplatin 80 mg/m^2^ (day 1), given every 4 weeks (MAP regimen). The comparator group included 16 women, who received standard NACT combination of paclitaxel 175 mg/m^2^ and carboplatin (6 AUC), given every 3 weeks (TCbP scheme).

**Results:**

None of the patients treated by the MAP scheme demonstrated complete pathologic response in ovaries, while 4 women showed absence of tumor cells in surgically excised omental specimens. When chemotherapy response scores (CRS) were considered, poor responsiveness (CRS 1) was not observed in the MAP group, but was common for the TCbP regimen (6/16 (38 %) for ovaries and 5/16 (31 %) for omentum; *p* = 0.05 and 0.12, respectively). Median treatment-free interval (TFI) was not reached in women treated by the MAP, but was 9.5 months for the TCbP scheme (*p* = 0.1). The rate of the recurrence within 1 year after the completion of the treatment was 4/10 (40 %) for the MAP and 10/13 (77 %) for the TCbP (*p* = 0.1).

**Conclusions:**

The attempt to intensify NACT by administering combination of 3 drugs did not result in high rate of complete pathologic responses. However, there was a trend towards higher efficacy of the MAP regimen versus conventional TCbP scheme with regard to CRS and clinical outcomes.

## Background

Ovarian cancer (OC) is a common malignancy, which holds the leading position in the mortality caused by gynecological tumors [1]. The worldwide incidence of OC approaches approximately three hundred thousand new cases per year, with almost two-thirds of affected patients dying from this disease [2]. High-grade serous ovarian cancer (HGSOC) is the most frequent OC histological type. A significant portion of HGSOCs is attributed to germ-line mutations in *BRCA1* or *BRCA2* genes. BRCA1/2-driven ovarian tumors usually develop via inactivation of the remaining allele of the involved gene. Consequently, these cancers demonstrate a tumor-selective deficiency in DNA repair by homologous recombination and pronounced sensitivity to platinum compounds, PARP inhibitors and mitomycin C [3, 4].

Ovarian tumors often do not cause symptoms at early stages; therefore, most HGSOC patients are diagnosed with already inoperable disease. These women are often subjected to neoadjuvant chemotherapy (NACT), which is aimed to reduce tumor burden and allow surgical intervention [5]. BRCA1-associated ovarian malignancies demonstrate significantly better responses to the NACT as compared to sporadic neoplasms [6]. Although these patients usually undergo complete cytoreductive surgery followed by adjuvant therapy, most BRCA1-driven HGSOCs eventually relapse [7]. These relapses are attributed to the acquisition of the resistance of tumor clones to systemic therapy. The most known mechanism of acquired platinum resistance is the emergence of mutations, which restore the open reading frame in the *BRCA1* gene [4]. This route is mainly applicable to heavily pretreated patients but appears to be less characteristic for the initial phases of OC therapy [8]. On the other hand, NACT often results in the selection of BRCA1-proficient cells, which exist in small amounts in chemonaive tumors and repopulate tumor mass during platinum exposure [9].

Intensification of the therapy is a common approach aimed to prevent the emergence of resistant clones. We have previously reported promising results of applying cisplatin plus mitomycin C combination for the NACT of BRCA1-driven carcinomas. This therapy resulted in a significant reduction of the tumor burden in all analyzed patients and in complete pathologic responses observed in 2/12 (17 %) treated women [10]. We reasoned that combining this regimen with an additional drug may further improve the outcomes of NACT. Previous studies suggested that BRCA1-driven tumors are particularly sensitive to anthracyclines, while their responsiveness to taxanes is under the question [10, 11]. Consequently, we decided to add doxorubicin to cisplatin plus mitomycin C as a third drug. Here we present the results of the trial involving this 3-drug combination.

## Methods

The design of the study was discussed on the council involving medical oncologists, cancer gynecologists and hereditary cancer specialists. It was decided that the pilot trial would include 10 patients with initially inoperable BRCA1-driven HGSOC, and the main end-point will be the rate of pathologic complete responses. While all patients received the same neoadjuvant regimen (MAP: mitomycin C 10 mg/m^2^ (day 1), doxorubicin 30 mg/m^2^ (days 1 and 8), cisplatin 80 mg/m^2^ (day 1), given every 4 weeks), the physicians were permitted to administer the therapy of their choice after the surgery. This approach provided some flexibility given that the combination of carboplatin and paclitaxel (TCbP) is a standard option for the treatment of ovarian cancer [1] and that some post-NACT tumor samples have restored BRCA1 function and therefore may not be potentially responsive to platinum drugs [8, 9]. The recruitment of patients was performed from August 2017 to December 2018 based on the results of the PCR-based test for Slavic recurrent germ-line mutations [12, 13]. According to the study protocol, all tumor samples were subjected to the loss-of-heterozygosity (LOH) analysis before the NACT and after the surgery. LOH test was performed as described in [9]. All tumors were also analyzed for the *TP53* somatic mutations, given that *TP53* inactivation is a ubiquitous feature of BRCA1-driven carcinomas [14]. The study was approved by the local Ethics Committee. All patients included in the study provided informed consent.

Although this study was not randomized, we considered for the comparison of treatment outcomes 16 consecutive patients with germ-line *BRCA1* mutations, who were referred to the N.N. Petrov Institute of Oncology (St.-Petersburg, Russia) between February 2017 and December 2019 and were subjected to a standard NACT combination of paclitaxel 175 mg/m^2^ plus carboplatin (6 AUC), given every 3 weeks. Most of these patients were negative for PCR-detectable recurrent *BRCA1* mutations; however, they were found to carry a germ-line pathogenic allele upon the analysis of the entire *BRCA1* and *BRCA2* coding sequence, i.e., after the start of NACT [13].

All women receiving MAP or TCbP were managed by the same surgical team. Tumor responses were evaluated according to RECIST criteria using computed tomography and magnetic resonance imaging. None of the patients treated by MAP or TCbP received bevacizumab. Three patients in the MAP arm but none in the TCbP group were subjected to the hyperthermic intraperitoneal chemotherapy (HIPEC) during surgery. None of the included patients described in this report received maintenance by PARP inhibitors after completion of the first-line therapy, as this indication was not approved in Russia at the time of the study.

The statistical analysis was performed using SPSS 13.0 software package. Age distribution and the duration of the follow-up were compared by the Mann-Whitney U-test. Median treatment-free interval (TFI) was evaluated using Kaplan-Meyer curves. Other comparisons were performed with the Fisher’s exact test.

## Results

Five patients included in the study of the neoadjuvant combination of mitomycin C, cisplatin and doxorubicin had stage IIIC HGSOC and another 5 women were diagnosed with stage IV disease (Table 1). Partial response to this therapy was observed in all 10 cases considered. Seven women had toxicities of grades 1 or 2; 2 patients had toxicity grade 3 and 1 subject experienced grade IV thrombocytopenia. None of the HGSOCs showed a complete pathological response in the ovaries, and only one woman demonstrated chemotherapy response score (CRS) 3, according to Böhm et al. [15]. Omental tumor response, which is more predictive for the disease outcome than adnexal CRS [15], showed considerably better values: 4 women had no residual tumor cells in the omentum, 1 patient had CRS 3 and 5 cases demonstrated CRS 2. There were no instances of poor responsiveness to the therapy categorized as CRS 1. The median TFI and progression-free survival (PFS) were not reached.

**Table 1:** BRCA1-mutated HGSOC patients receiving neoadjuvant therapy consisting of mitomycin C, doxorubicin and cisplatin

ID	Age	Stage	*BRCA1* mutation	Somatic *BRCA1* status before NACT	MAP cycles (NACT)	Surgical debulking	Response by RECIST	CRS (ovary / omentum)	Somatic *BRCA1* status after NACT	ACT (cycles)	TFI, months	PFS, months	OS, months	Toxicities and grades	TP53 mutation
MAP1	64	IIIC	c.5266dupC	LOH	3	Complete + HIPEC	PR (-44 %)	CRS 3 / no tumor cells in omentum	LOH	MAP (2)	25.4+	32.2+	32.2+	Anemia 1; nephrotoxicity 1	C135W
MAP2	35	IVA (pleuritis)	c.68_69delAG	LOH	3	Complete + HIPEC	PR (-73 %)	CRS 2 / no tumor cells in omentum	na	MAP (1), AT (2)	6.9	14.4	26.9	Anemia 1	R175H
MAP3	50	IIIC	c.4034delA	LOH	4	Complete	PR (-35 %)	CRS 2 / CRS 2	LOH	MAP (3)	23.6+	31.6+	31.6+	Anemia 1	Y234H
MAP4	57	IVB (lymph nodes)	c.5266dupC	LOH	3	Complete + HIPEC	PR (-90 %)	CRS 2 / CRS 3	Retention of the wild-type allele	AT (3)	29.2+	36.6+	36.6+	Diarrhea 1; emesis 1; gastritis 1; nausea 1; leukopenia 2; nephrotoxicity 2; thrombocytopenia 4	R213X
MAP5	50	IIIC	c.5266dupC	na	3	Complete	PR (-33 %)	CRS 2 / no tumor cells in omentum	LOH	MAP (3)	22.2+	29.5+	29.5+	Anemia 1; leukopenia 1; nausea 1; thrombocytopenia 1	c.97-1G > A
MAP6	45	IVB (spleen, lymph nodes)	c.1961delA	LOH	4	Suboptimal	PR (-47 %)	CRS 2 / CRS 2	LOH	TCbP (6)	21+	30.4+	30.4+	Anemia 1; nausea 1; thrombosis	R213X
MAP7	56	IVB (pleuritis, lymph nodes)	c.5266dupC	na	3	Complete	PR (-47 %)	CRS 2 / CRS 2	LOH	MAP (1)	4.1	8.3	29.8+	Anemia 2; nephrotoxicity 3	c.314delG
MAP8	58	IIIC	c.4034delA	LOH	5	Complete	PR (-46 %)	CRS 2 / CRS 2	LOH	TCbP (4)	11.5	20.7	28.2+	Anemia 1; neutropenia 2	R248Q
MAP9	46	IIIC	c.4034delA	LOH	4	Complete	PR (-64 %)	CRS 2 / CRS 2	LOH	MAP (2)	3.1	11.2	15.8	Thrombocytopenia 2	I255S
MAP10	40	IVB (pleuritis, lymph nodes)	c.5266dupC	LOH	4	Complete	PR (-36 %)	na / no tumor cells in omentum	na	None	28.5+	37.6+	37.6+	Anemia 1; hepatotoxicity 2; thrombocytopenia 2; nephrotoxicity 3	M133R

*ACT* Adjuvant chemotherapy, *AT* Doxorubicin 60 mg/m^2^ and paclitaxel 175 mg/m^2^, every 3 weeks, *CRS* Chemotherapy response score, *HIPEC* hyperthermic intraperitoneal chemotherapy, *LOH* Loss of heterozygosity, *na* Not analyzed, *MAP* Mitomycin C 10 mg/m^2^ (day 1), doxorubicin 30 mg/m^2^ (days 1 and 8) and cisplatin 80 mg/m^2^ (day 1), given every 4 weeks, *NACT* Neoadjuvant chemotherapy, *OS* Overall survival, *PFS* Progression-free survival, *PR* Partial response, *TCbP* Paclitaxel 175 mg/m^2^ plus carboplatin (6 AUC), given every 3 weeks, *TFI* Treatment-free interval.

Notes: Tumor responses presented in the table describe the status of patients observed after the completion of the NACT, i.e. straight before the surgery. Patient MAP10 was diagnosed with ovarian cancer upon surgery, which was performed in another hospital and was limited to the excision of ovaries. She was considered eligible for the NACT study, as she had a significant tumor burden and could not be subjected to primary debulking surgery; she received no adjuvant therapy, as no residual tumor cells was seen in the surgical material. Patient MAP4 demonstrated the restoration of heterozygosity in a post-NACT tumor sample, suggesting that the tumor may no longer be platinum-sensitive [8, 9]; based on this finding, combination of paclitaxel and doxorubicin was given after the surgery; similarly, this combination was incorporated in the adjuvant treatment for patient MAP2, where the molecular analysis of post-NACT tumor tissue failed to establish somatic BRCA1 status. Patients MAP6 and MAP8 received TCbP combination after the surgery due to preference of their primary physicians.

**Fig. 1:**
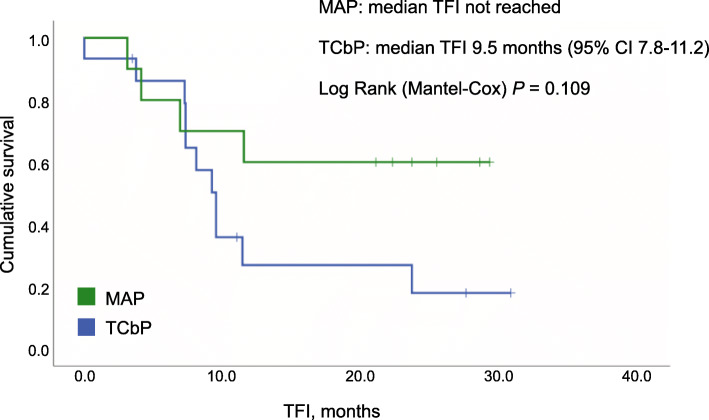
Treatment-free interval for patients treated with the combination of mitomycin C, cisplatin and doxorubicin and for women treated by the paclitaxel plus carboplatin doublet

Sixteen patients receiving paclitaxel plus carboplatin had slightly more favorable stage distribution, as 12 subjects had HGSOC of stage IIIC and 4 patients demonstrated stage IV disease (Table 2). While all patients treated by MAP showed partial response, 4/16 (25 %) women subjected to the TCbP combination produced only the disease stabilization and there was one HGSOC with the progression on this therapy. There was a remarkable difference from MAP regimen with regard to pathological responses, as minimal response score was observed in 6/16 (38 %) cases for ovarian tumor masses and 5/16 (31 %) HGSOCs for omental metastases (*p* = 0.05 and 0.12, respectively). While the median follow-up for the TCbP group was shorter than for MAP patients, median TFI was already achieved and reached 9.5 months (Fig. 1). Thirteen patients had sufficient follow-up to evaluate 1-year outcomes; the recurrence rate at 1 year after the completion of the treatment was 10/13 (77 %) for the TCbP, while the same value was 4/10 (40 %) for the MAP regimen (*p* = 0.1).

**Table 2:** Comparative characteristics of patients with BRCA1-mutated HGSOC receiving NACT combination of mitomycin C, doxorubicin and cisplatin versus women treated by paclitaxel plus carboplatin

Clinical characteristics	MAP group (*N* = 10)	TCbP group (*N* = 16)	Statistical comparison
Median age of onset (range)	50 (35–64)	49 (37–72)	Not significant
Pattern of *BRCA1* mutations	c.5266dupC (*n* = 5), c.4034delA (*n* = 3), c.68_69delAG (*n* = 1), c.1961delA (*n* = 1)	c.5266dupC (*n* = 2), c.4034delA (*n* = 2), c.68_69delAG (*n* = 1), C61G (*n* = 1), c.1510delC (*n* = 1), Q563X (*n* = 1), c.2076dupT (*n* = 1), c.2983_2984delAA (*n* = 1), c.3247del5 (*n* = 1), c.3601_3602delGG (*n* = 1), c.3718_3719delCA (*n* = 1), Y1509X (*n* = 1), G1706E (*n* = 1), c.5152 + 1G > T (*n* = 1)	*p* = 0.005 (founder vs. non-founder mutations; Fisher’s exact test)
FIGO stage			
IIIC	5 (50 %)	12 (75 %)	*p* = 0.23 (stage III vs. IV; Fisher’s exact test)
IVA	1 (10 %)	2 (13 %)	
IVB	4 (40 %)	2 (13 %)	
NACT cycles (range)	3–5	3–8	
Cytoreduction			
Optimal	9 (90 %)	14 (88 %)	*p* = 1.0 (Fisher’s exact test)
Suboptimal	1 (10 %)	1 (6 %)	
None	0	1 (6 %)	
Response by RECIST			
CR	0	0	*p* = 0.12 (objective response vs. lack of objective response; Fisher’s exact test)
PR	10 (100 %)	11 (69 %)	
SD	0	4 (25 %)	
PD	0	1 (6 %)	
Chemotherapy response score (CRS) in the ovaries			
CRS 1	0	6 (38 %)	*p* = 0.05 (CRS 1 vs. other; Fisher’s exact test)
CRS 2	8 (80 %)	9 (56 %)	
CRS 3	1 (10 %)	0	
Tissue not available for evaluation	1 (10 %)	1 (10 %)	
Chemotherapy response score (CRS) in the omentum			
CRS 1	0	5 (31 %)	*p* = 0.12 (CRS 1 vs. other; Fisher’s exact test)
CRS 2	5 (40 %)	8 (50 %)	
CRS 3	1 (10 %)	0	
No tumor cells	4 (40 %)	2 (13 %)	
Tissue not available for evaluation	0	1 (6 %)	
ACT cycles (range)	1–6	1–6	
Median follow-up, months (range)	30.1 (15.8–36.6)	23.4 (10.7–45.2)	*p =* 0.28 (Mann-Whitney Test)
Median treatment-free interval (95 % CI)	Not reached	9.5 (7.8–11.2)	*p* = 0.109 (Log Rank [Mantel-Cox])
Recurrence within one year after completion of treatment	4 (40 %)	10/13^a^ (77 %)	*p* = 0.1 (Fisher’s exact test)

*ACT* adjuvant chemotherapy, *CRS* chemotherapy response score, *MAP* mitomycin C 10 mg/m^2^ (day 1), doxorubicin 30 mg/m^2^ (days 1 and 8), cisplatin 80 mg/m^2^ (day 1), given every 4 weeks, *NACT* neoadjuvant chemotherapy, *TCbP* paclitaxel 175 mg/m^2^ plus carboplatin (6 AUC), given every 3 weeks

^a^13 out of 16 patients had sufficient follow-up for the estimation of 1-year recurrence rate

## Discussion

Our previous study involving 12 BRCA1-mutated HGSOCs treated with the combination of cisplatin and mitomycin C revealed complete pathologic responses in 2 out of 12 patients [10]. We anticipated that the addition of doxorubicin to this combination may increase the rate of elimination of all tumor cells detectable in surgically excised tissues. The obtained data are sufficient to state that the applied triplet does not significantly increase the rate of complete pathologic responses as compared to the previously applied combination of two drugs.

At the same time, short-term results of MAP therapy look encouraging. In addition to a reasonably good rate of objective responses, half of the included cases demonstrated complete or nearly-complete absence of tumor cells in the omentum. Omental response score is the main predictor of the long-term outcomes of NACT, so it is a valuable marker allowing robust evaluation of various chemotherapy regimens [15].

Previous studies suggested that the TCbP regimen may be less efficient in BRCA1-mutated HGSOCs as compared to other NACT schemes [10]. These data sets compared prospective and retrospective patients treated by different surgeons. The quality of surgical debulking is critical for the outcome of HGSOC treatment, therefore these comparisons are prone to biases. Within the present study, we analyzed groups of patients who were managed at the same time interval by the same group of surgeons. However, the MAP and TCbP groups of patients were not balanced with regard to the pattern of mutations. The selection of patients for the MAP therapy involved rapid PCR tests for recurrent Slavic mutations [13]. The TCbP HGSOC group is significantly enriched by subjects with “rare” *BRCA1* pathogenic alleles, which were detected by the next-generation sequencing analysis after the start of NACT. There are some data suggesting that distinct *BRCA1* and *BRCA2* mutations may exert distinct sensitivity to platinum compounds and PARP inhibitors [4]. Although we acknowledge differences in the pattern of *BRCA1* mutations as a limitation of the study, it should be noted that all published trials on PARP inhibitors did not consider the type of mutation as a confounding factor [4, 16].

The landscape of the treatment BRCA1/2-mutated HGSOC is rapidly evolving. In particular, PARP inhibitors have been recently included in the standards for the first-line maintenance therapy, as they significantly delay the time to tumor recurrence [16]. None of the patients considered in this report received PARP inhibitors because they were not locally approved for early lines of HGSOC treatment at the time of the study. Consequently, it is unclear whether the differences observed between distinct NACT regimens will be maintained upon the incorporation of PARP-targeted drugs.

## Conclusions

In summary, this study suggests that BRCA1-associated HGSOCs may require distinct therapeutic NACT regimens as compared to conventional TCbP doublet. If this is the case, the fast turn-around time for *BRCA1/2* testing could become a critical factor for appropriate treatment decisions. Recent data indicate that BRCA1/2-associated HGSOCs do not show inferior outcomes when treated by NACT before the surgery, while primary surgical intervention is clearly the best approach in sporadic ovarian tumors [7, 17, 18]. These findings are likely to increase the acceptance of NACT for *BRCA1/2* germline mutation carriers and, therefore, stimulate large neoadjuvant clinical trials for this category of HGSOC patients.

## Data Availability

The datasets used and/or analyzed during the current study are available from the corresponding author on reasonable request.

## Abbreviations

CRS: Chemotherapy response score
HGSOC: High-grade serous ovarian cancer
LOH: Loss of heterozygosity
MAP: Mitomycin C, anthracycline, platinum
NACT: Neoadjuvant chemotherapy
OC: Ovarian cancer
PFS: Progression-free survival
TCbP: Taxanes and carboplatin
TFI: Treatment-free interval
